# Two coding-complete genomes of porcine reproductive and respiratory syndrome virus 2 (PRRSV-2) from field clinical samples in the Philippines

**DOI:** 10.1128/mra.01164-24

**Published:** 2025-01-08

**Authors:** Andrew Montecillo, Jimwel Bryan Christopher Ferrer, Zyne Baybay, Ruffa Maze Balmes, Roselle Falconite-Cudal, Mary Grace Alba, Karl Alexander Fabros, Timothy James Dela Paz, Lucille C. Villegas, Wreahlen Cariaso, Homer Pantua

**Affiliations:** 1 Institute of Biological Sciences, College of Arts and Sciences, University of the Philippines Los Baños, Los Baños, Laguna, Philippines; 2 BioAssets Corporation, Santo Tomas, Batangas, Philippines; 3 ELANCO Philippines Inc., Mandaluyong City, Philippines; DOE Joint Genome Institute, Berkeley, California, USA

**Keywords:** porcine reproductive and respiratory syndrome virus, PRRSV-2, nanopore sequencing, coding-complete genomes

## Abstract

We report two coding-complete genome sequences of porcine reproductive and respiratory syndrome virus 2 from field clinical samples obtained in 2021 (BA2021012A) and 2023 (ME20230008B-2) from the Philippines. BA2021012A (15,388 bp) is classified as a lineage L8C strain while ME20230008B-2 (15,513 bp) is a vaccine-like strain in lineage L7.

## ANNOUNCEMENT

Porcine reproductive and respiratory syndrome (PRRS), first described in the United States in the late 1980s and in Europe in the early 1990s, is a serious global infectious disease of the swine industry. The disease is caused by the PRRS virus (genus *Betaarterivirus*)—a positive single-stranded RNA virus from the family *Arteriviridae* (1). There are two species of PRRSV (2, 3), namely, *Betaarterivirus europensis* (PRRSV-1 or the European strain) and *Betaarterivirus americense* (PRRSV-2 or the North American strain), both causing similar clinical symptoms and whose genomes share only approximately 60% nucleotide identity. Major outbreaks of the disease in the Philippines were first reported in 2007 (4) and have been causing extensive losses estimated at $138 million annually (5).

Two field clinical samples were obtained, one serum sample in September 2023 from Mindanao, Philippines, from nursery pigs demonstrating respiratory signs in starter-growers (sample code: ME20230008B-2), and lung tissue samples obtained in 2021 from a farm in Sariaya, Quezon province, from farrowing pigs showing both reproductive and respiratory symptoms (sample code: BA2021012A). Samples were collected by licensed veterinary consultants following the guidelines of the Bureau of Animal Industry, Department of Agriculture (Philippines), and were submitted to BioAssets Veterinary Research and Diagnostic Laboratory (Philippines) for processing.

The total nucleic acids were extracted from the samples using the IndiSpin Pathogen Kit (Indical Bioscience GmbH, Leipzig, Germany) and were tested by quantitative reverse transcription PCR (RT-qPCR) using the virotype PRRSV RT-qPCR kit (Indical Bioscience GmbH, Leipzig, Germany). Full-length ORF5 (envelope protein) and genomes from confirmed PRRSV-2-positive samples were sequenced following the targeted amplicon sequencing and long amplicon tiling sequencing protocols (6), respectively. Library was prepared using the Native Barcoding Kit expansion (LSK-109 and EXP-NBD104; Oxford Nanopore Technologies, England, UK) and sequenced on MinION SpotON R9.4.1. FLO-MIN106 flow cell (ONT) using MinKnow Software (v.24.02.6; ONT). Reads were basecalled using the super accurate model, and adapters were trimmed in MinKnow Software with default parameters. Reads were filtered in filtlong (https://github.com/rrwick/Filtlong) based on quality (--keep_percent 90). ORF5 and whole genomes were assembled using Medaka v.2.0.1 (ONT), SAMtools v.1.21 (7), and BCFtools v.1.14 (8) as described by Caserta et al. (6). ISU PRRSVIEW Lineage and Vaccine Tool were employed to determine the PRRSV-2 lineage (9, 10). Extraction, library preparation, and sequencing were performed following the manufacturer’s protocol. Blastn (11) was used in sequence similarity analysis.

For full-length ORF5 (603 bp) sequencing, 11,844 reads (mean depth of 11,300×) were generated for ME20230008B-2 and 18,125 reads (mean depth of 17,300×) for BA2021012A. ME20230008B-2 belongs to the L7 lineage with 99.8% nucleotide identity with Prime Pac PRRS RR (Merck), a vaccine strain, while BA2021012A belongs to L8C lineage and is considered a field strain.

**Fig 1:**
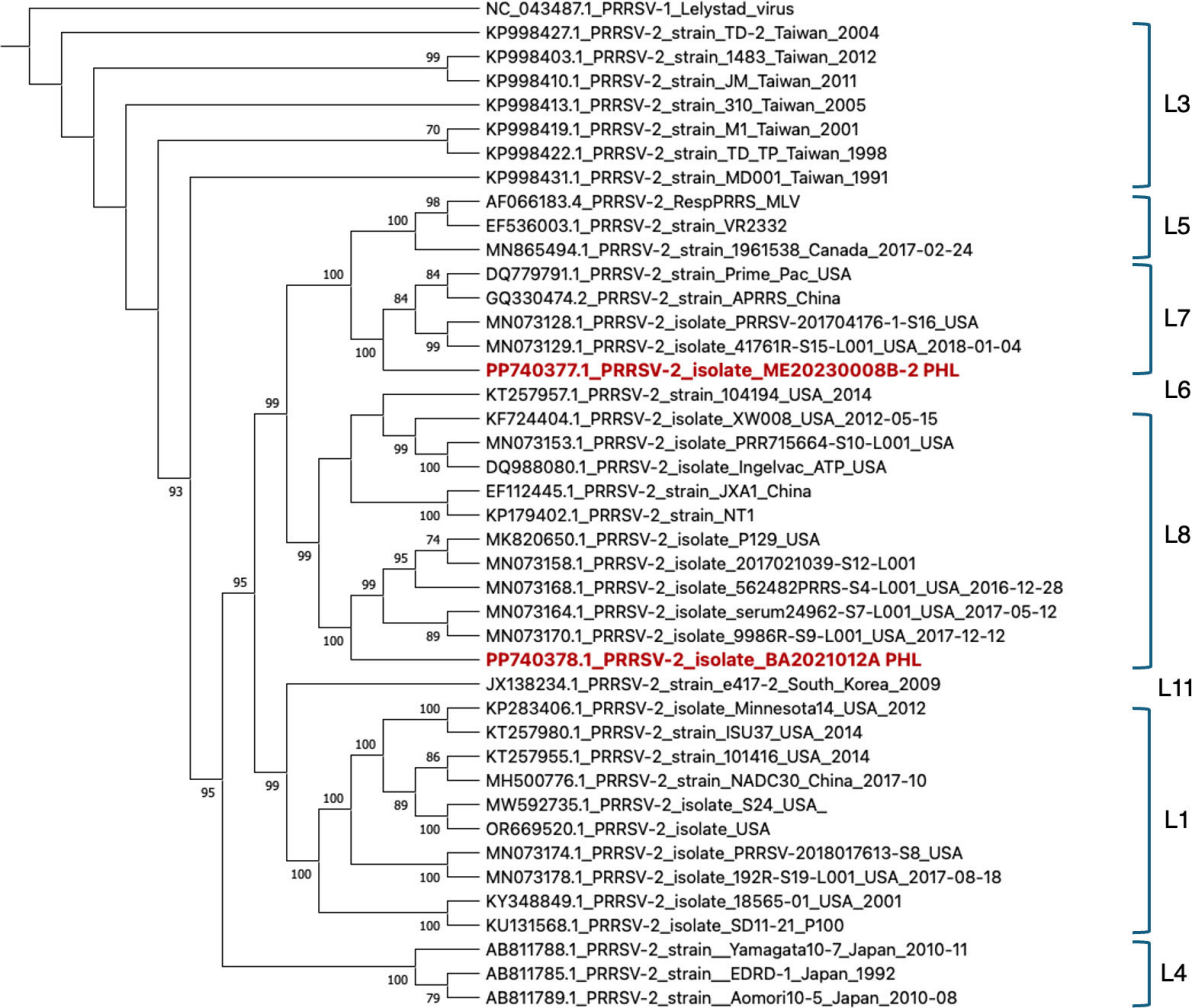
Whole-genome maximum-likelihood consensus tree of select PRRSV reference genomes and genomes of Philippines ME20230008B-2 (PP740377.1) and BA2021012A (PP740378.1) strains inferred using the ultrafast bootstrap implemented in the IQ-TREE software v.2.3.6 (12) with substitution model: GTR+F+I+R3, determined using the built-in ModelFinder with -m MFP option, after whole-genome alignment in MAFFT v. 5.20 (13) with gap opening penalty of 1.53. Only the 5′- and 3′-ends of the alignment were trimmed. The tree is rooted at NC_043487.1 as outgroup. The bootstrap resampling (1,000 replications) support values (>70%) are shown at the nodes. Lineage classification of PRRSV-2 genomes (L1 to L11, as appropriate) are indicated by square brackets. The tree is visualized in MEGA X (14).

Whole-genome sequencing statistics are summarized in Table 1. Based on BLASTn search (*nt* database, accessed 15 July 2024), ME20230008B-2 is most similar (99.92%) to PRRSV isolate 41,761 R-S15-L001 (MN073129.1), a 2018 lineage 7 strain from the USA, while BA2021012A is most similar (98.36%) to PRRSV isolate 2017021039-S12-L001 (MN073158.1), a 2017 Lineage 8C strain from the USA (Fig. 1).

**TABLE 1:** Long-read whole-genome sequencing summary statistics of ME20230008B-2 and BA2021012A

Strain	Total reads generated	Reads N50 (bp)	Mean coverage (×)	Assembly size (bp)	Number of contig	%GC
ME20230008B-2	143,864	1,445	3,500	15,513	1	52.7
BA2021012A	143,313	783	2,180	15,388	1	52.76

## Data Availability

The full-length ORF5 sequences are deposited in the NCBI GenBank as PQ381624.1 for ME20230008B-2 and PQ381625.1 for BA2021012A. The genome sequences of ME20230008B-2 and BA2021012A are deposited in the NCBI GenBank under accession numbers PP740377.1 and PP740378.1, respectively. Raw sequence data for the full-length ORF5 and whole genome for both ME20230008B-2 and BA2021012A are available from GenBank SRA under BioProject accession no. PRJNA1177992 as SRX26569532 and SRX26569533 for the ORF5 and SRX26507955 and SRX26507956 for the whole genomes. The version described in this paper is the first version.
